# 3D‐Printed Cut Guides and Custom Prostheses for Pelvic Reconstruction in Bone Sarcoma Patients: Complications, Survival Rates, and Functional Outcomes

**DOI:** 10.1111/os.70192

**Published:** 2025-12-29

**Authors:** Luca Cevolani, Benedetta Spazzoli, Eric Lodewijk Staals, Costantino Errani, Laura Campanacci, Paolo Spinnato, Massimiliano De Paolis, Giuseppe Bianchi, Davide Maria Donati

**Affiliations:** ^1^ Unit of 3rd Orthopaedic and Traumatologic Clinic Prevalently Oncologic IRCCS Istituto Ortopedico Rizzoli Bologna Italy; ^2^ Diagnostic and Interventional Radiology IRCCS Istituto Ortopedico Rizzoli Bologna Italy; ^3^ Department of Orthopaedics, Istituto di Ricerca e Cura a Carattere Scientifico (IRCCS) Azienda Ospedaliera Universitaria di Bologna Bologna Italy; ^4^ Department of Biomedical and Neuromotor Sciences (DIBINEM) Alma Mater Studiorum University of Bologna Bologna Italy

**Keywords:** 3D‐printed custom‐made cut guide, 3D‐printed custom‐made prosthesis, bone sarcoma, pelvic reconstruction

## Abstract

**Introduction:**

Pelvic reconstruction following bone sarcoma resection presents significant challenges. This study evaluates the outcomes of using 3D‐printed custom‐made prostheses and cutting guides to improve surgical precision and functional results in periacetabular reconstructions. Therefore, in this study, we asked: (1) What is the cumulative incidence of reoperation for any reason following pelvic resection and reconstruction with a custom‐made 3D‐printed prosthesis involving the acetabulum in patients with primary bone sarcoma, and what factors contribute to an increased risk of reconstruction failure? (2) Does the use of 3D custom‐made cutting guides, combined with a 3D custom‐made hemipelvis prosthesis, ensure the attainment of safe resection margins and allow for anatomical reconstruction with optimal fit at the bone‐prosthesis interface? (3) What were the observed outcome scores as measured by the Musculoskeletal Tumor Society (MSTS) Score? Additionally, how do the type of resection and the volume of the primary bone sarcoma affect the outcomes in relation to the type of reconstruction?

**Materials and Methods:**

We conducted a retrospective review of 24 patients treated for primary bone sarcomas at our institution from January 2013 to December 2023. Each patient received a 3D‐printed cutting guide and a 3D‐printed custom‐made prosthesis tailored to their specific anatomical needs, based on high‐resolution imaging and computer‐aided design.

**Results:**

The use of custom‐made 3D prostheses resulted in a reoperation rate of 46%, primarily due to complications such as infection and mechanical failures. Specific complications included an 8% rate of deep infections and mechanical issues like aseptic loosening. Local recurrence was observed in 5 patients (21%) at a median time of 5 months post‐surgery. Despite these challenges, the average MSTS score was 83.7%, indicating a high level of functional recovery post‐surgery.

**Conclusions:**

The integration of 3D printing in pelvic reconstructions for bone sarcomas significantly enhances anatomical and functional outcomes. However, the technology demands further refinement to reduce complication rates. Continued advancements in 3D‐printing materials and techniques are crucial to maximizing the benefits of this innovative approach in orthopedic oncology.

## Graphical abstract

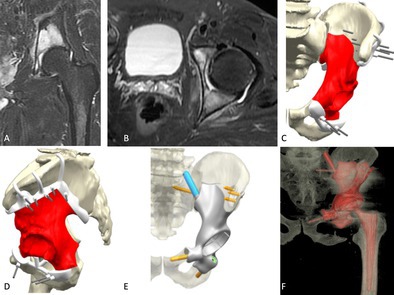

## 1 Introduction

Although the pelvis is a rare site for primary bone sarcoma, with an incidence of 5%–10% of all malignant bone tumors [1], when the periacetabular region is involved, hip reconstruction following bone resection presents some of the most difficult challenges for musculoskeletal oncologists in terms of reconstruction longevity, joint function preservation, and obtaining wide tumor resection margins [2, 3]. Advances in neoadjuvant therapies and preoperative imaging techniques have made limb‐salvage surgery more feasible, reducing the need for hindquarter amputations in cases of unresectable tumors involving neurovascular structures [4].

Image processing and 3D printing have enabled the possibility of performing bone resections and reconstructing bone defects with personalized, custom‐made cut guides and pelvic prostheses [5]. 3D‐printed cut guides offer potential advantages in obtaining adequate surgical margins compared to intraoperative navigation systems, which are limited by cost, a steep learning curve, and a time‐consuming registration procedure. Augmented reality systems are still in an experimental phase [6]. Moreover, there is no consensus on the best reconstruction technique. The main challenges in pelvic reconstruction are achieving good primary stability of the implant and ensuring progressive integration at the prosthesis–bone interface [7]. The use of massive allografts [8, 9], allograft composite prostheses [10, 11], autografts [12], and hemi‐pelvic prostheses [13, 14] is often complicated by loosening, dislocation, infection, and nonunion. The porous surface of a 3D‐printed prosthesis ensures accurate matching with the resected bone surface and stimulates osteointegration between the implant and the host bone, while the accurate modeling of the prosthesis allows for the restoration of pelvic anatomy and function [15, 16, 17]. However, little is known about the long‐term durability of these implants.

Therefore, in this study, we asked: (1) What is the cumulative incidence of reoperation for any reason following pelvic resection and reconstruction with a custom‐made 3D‐printed prosthesis involving the acetabulum in patients with primary bone sarcoma, and what factors contribute to an increased risk of reconstruction failure? (2) Does the use of 3D custom‐made cutting guides, combined with a 3D custom‐made hemipelvis prosthesis, ensure the attainment of safe resection margins and allow for anatomical reconstruction with optimal fit at the bone–prosthesis interface? (3) What were the observed outcome scores as measured by the Musculoskeletal Tumor Society (MSTS) Score? Additionally, how do the type of resection and the volume of the primary bone sarcoma affect the outcomes in relation to the type of reconstruction?

## 2 Material and Methods

### 2.1 Study Design and Setting

We conducted a retrospective study analyzing patients who underwent pelvic reconstruction with a 3D custom‐made prosthesis involving the periacetabular area after primary bone sarcoma resection, at a single institution, from January 2013 to December 2023. The inclusion criteria were as follows: patients with primary bone tumor of the pelvis affecting the acetabular area, who had a follow‐up of at least 6 months or until death, and whose resections were performed using a 3D‐printed custom cutting guide. Exclusion criteria were patients who underwent surgeries other than resection for primary sarcoma, those treated for solitary metastasis of the pelvis, or those with previous bone loss due to osteomyelitis or prosthesis removal.

### 2.2 Participants

We collected data from 24 patients who met the inclusion criteria. The demographic and clinical information gathered included the patients' age at the time of surgery, gender, diagnosis, disease stage, location and size of the bone lesion, functional status, as well as any adverse reactions, side effects, and follow‐up details. Descriptive characteristics of the patients are reported in Table 1. The mean age at the time of surgery was 42.5 ± 15.3 years. The mean follow‐up was 60 ± 41 months.

**TABLE 1:** Characteristics of patients undergoing hemipelvis replacement surgery.

Characteristic	All patients (*N* = 24)
Gender	
Male	16 (66.7%)
Female	8 (33.3%)
Age (years)	42.5 ± 15.3
Follow‐up (months)	60 ± 41
Tumor histology	
Osteosarcoma	4 (16.7%)
Ewing’ sarcoma	3 (12.5%)
Chondrosarcoma grade 2	13 (54.2%)
Chondrosarcoma grade 3	2 (8.3%)
Aggressive osteoblastoma	2 (8.3%)
Neoadjuvant chemotherapy	9 (37.5%)
Type of resection according^a^	
Type II	4 (16.7%)
Type I + II	4 (16.7%)
Type II + III	12 (50%)
Type I + II + III	4 (16.7%)
Oncologic status at the end of follow‐up	
NED	20 (83.3%)
AWD	1 (4.2%)
DOD	3 (12.5%)

Abbreviations: AWD = alive with disease; DOD = dead for progressive disease; NED = no evidence of disease.

^a^ According to the Enneking‐Dunham Classification system [18].

### 2.3 Descriptive Data

There were 4 patients who presented with osteosarcoma, 3 with Ewing sarcoma, 13 with chondrosarcoma Grade 2, 2 with chondrosarcoma Grade 3, and 2 with aggressive osteoblastoma. All nine patients with malignant sarcoma underwent pre‐ and postoperative chemotherapy; none received pre‐ or postoperative radiotherapy. The type of pelvic resection was classified according to the system proposed by Enneking and Dunham [18], as follows: PI (ilium), PII (periacetabular), PIII (pubic rami), and PIV (sacrum). Surgical margins were evaluated on the basis of surgical and pathological reports according to the system described by Enneking [19].

### 2.4 Preoperative Planning

A 3D model of the pelvic bone was generated from CT scans with slice thickness less than 2 mm. Concurrently, the 3D model of the tumor was segmented from MRI data using the ITK‐Snap Insight segmentation and registration toolkit, which includes Snake automatic partitioning (Penn Image Computing and Science Laboratory, University of Pennsylvania) [20]. The alignment of CT‐based and MRI‐based models was carried out using Geomagic software (Geomagic Freeform; 3D Systems Inc.). The same surgical team (DD, BS) planned the tumor resections for all patients, focusing on the location and orientation of the osteotomy cuts. Their goal was to ensure both thorough tumor removal and enhanced contact between the host bone and the prosthesis, thereby optimizing osteointegration. To ensure oncological safety, the planned bone resection includes a minimum margin of 2 cm from the tumor. This precaution accounts for the potential growth of the lesion during the interval between preoperative planning and surgical intervention, in order to avoid an inadequate resection.

**FIGURE 1:**
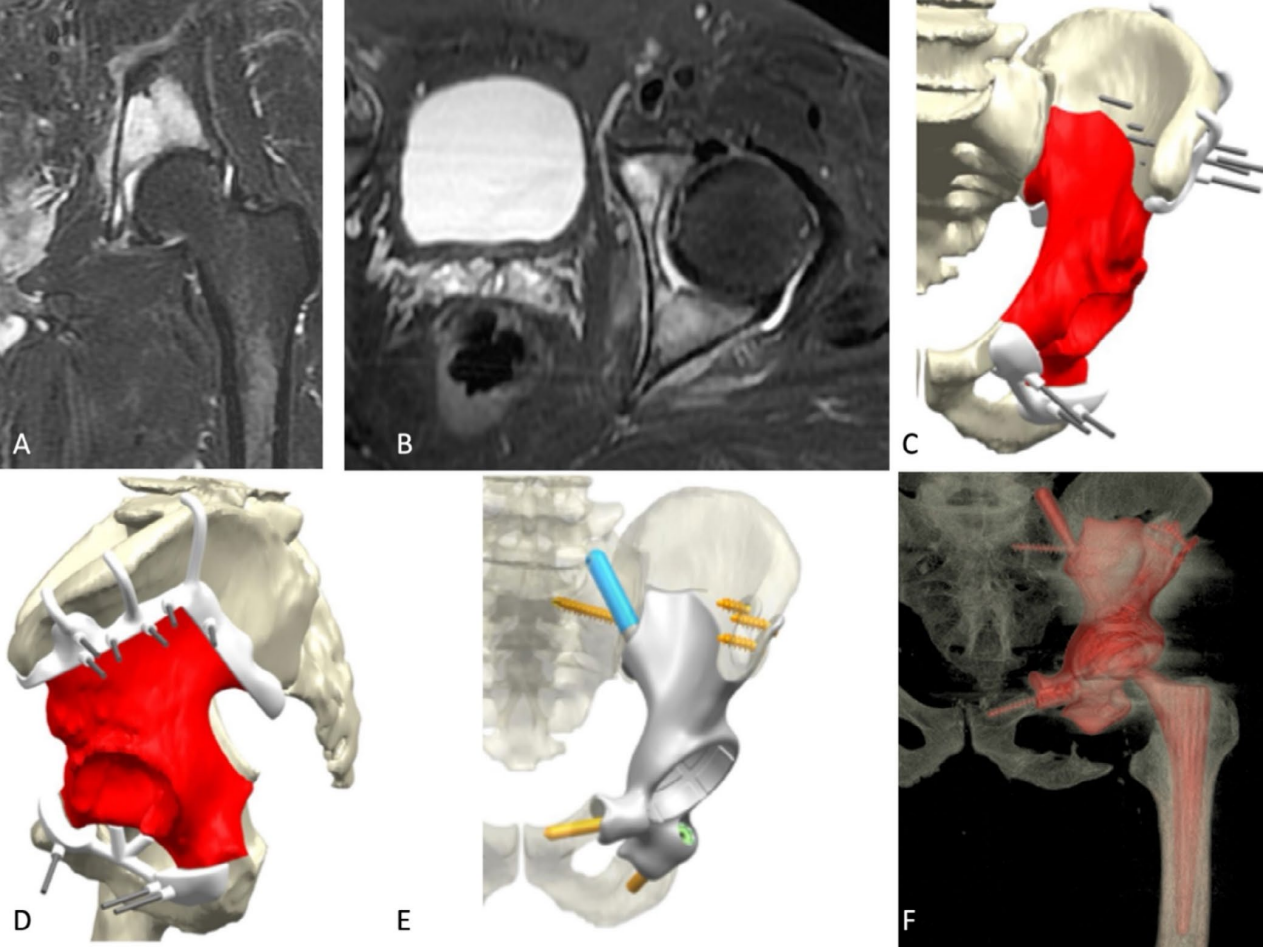
A 60‐year‐old male patient with Grade 2 chondrosarcoma. High‐intensity signals were observed in the coronal (A) and transverse (B) MRI images (T1‐fat suppression with contrast) in the left hemi‐pelvis, involving the periacetabular area but without infiltration into the hip joint. 3D computer renderings for the frontal (C) and lateral (D) views of the left hemi‐pelvis show the planned P1–P2–P3 resection highlighted [in red] and the planned 3D‐printed custom‐made cutting guide positioning and fixation [in white]. (E) 3D computer renderings post‐tumor resection depict the insertion of the 3D‐printed prosthesis and its fixation points. The actual cutting points were confirmed using a 3D‐printed bone model. The fixation points for the cancellous screws and stems were also determined, allowing for the calculation of the appropriate lengths for drilling and the sizes of the planned screws or stems. (E) Postoperative CT 3D reconstruction after the tumor resection and prosthetic reconstruction. It should be noted that the screw on the ischial branch is not included in the figure, as resection was extended intraoperatively and fixation omitted in that region, following soft tissue disease progression observed during chemotherapy.

The surgical model was sent to the prosthesis engineering team for printing, both as the cutting guide and as the custom‐made titanium prosthesis. The cutting guide was printed to match the exact shape of the bone, ensuring it could be positioned uniquely on the host bone (Figure 1). Although no formal 3D deviation analysis was performed between the virtual plan and the achieved osteotomy, the guide positioning was validated using printed anatomical bone models, and intraoperative adjustments were based on preoperative simulations. Minor discrepancies due to tumor extension or asymmetry were compensated through flexible adaptation of the implant design, allowing for optimal contact with the residual bone. These steps, although not quantified numerically, follow principles similar to those proposed in ICP‐based validation workflows [21] and contribute to ensuring surgical accuracy despite anatomical variability.

### 2.5 Surgical Technique

The patient was placed in a semi‐lateral decubitus position, with the ipsilateral hip elevated. An inverted “J” incision is made on the skin, beginning 5 cm ahead of the posterior–superior iliac spine and tracing along the iliac crest toward the anterior‐superior iliac spine. Once reaching the anterior–superior iliac spine, the incision continued along the lateral third of the inguinal ligament, extending approximately 10–15 cm down into the adductor region of the thigh.

After the iliac crest is exposed, the interval between the abdominal and gluteal muscles is developed to reveal the external surface of the iliac wing. Medially, the abdominal musculature must be carefully dissected from the iliac crest. The inguinal ligament is then detached from the anterior–superior iliac spine and reflected proximally. Subsequently, once the iliopsoas and the femoral bundle have been identified and protected, the pubic symphysis is exposed, and the adductor and pectineus muscles are detached from their insertions on the pelvis.

The cutting guides are designed using stable and easily identifiable bony landmarks—such as the iliac crest, anterior superior iliac spine, and acetabular margin—to ensure precise and reproducible positioning. These anatomical reference points must be carefully exposed through meticulous soft tissue dissection prior to guide placement. The three‐dimensional geometry of each guide is engineered to fit uniquely onto the host bone, eliminating the possibility of misalignment. Additionally, the guide's design is optimized to avoid interference with surrounding soft tissue masses, even in cases of large tumor volume.

During surgery, the guide fits precisely onto the bone surface by conforming to the landmarks defined during preoperative planning. This ensures accurate replication of the planned osteotomy. Once correctly positioned, the guide is secured to the bone with cortical screws, providing full stability throughout the cutting procedure.

The pubic branch guide is utilized in type II resections, but is not employed in type III resections, where the specimen is detached at the level of the pubic symphysis. Upon completion of the osteotomy, the specimen is removed, and the anatomy of the pelvic ring is reconstructed using a patient‐specific custom‐made prosthesis. A standard femoral stem is then implanted to complete the hip replacement [5].

To ensure a precise fit between the host bone and the prosthesis, both the osteotomy planes and implant geometry were planned with a tolerance not exceeding 1 mm. All patients underwent a postoperative CT scan of the hip to assess the accuracy of the bone osteotomy and the fit of the prosthesis.

To assess conformity to the preoperative surgical planning, we relied on three main criteria: histopathological confirmation of wide resection margins, absence of gaps greater than 1 mm between the bone and the prosthesis on postoperative CT imaging, and radiological assessment consistent with the absence of macroscopic residual disease in the host bone on CT scans.

In the postoperative period, a hip cast was applied for 6 weeks. Subsequently, the patient commenced the rehabilitation program. Protected weight bearing with crutches was permitted 45 days after surgery, alongside protected and assisted hip mobilization.

### 2.6 Follow‐Up

Clinical and radiological outcomes were evaluated during outpatient visits at 45 days postoperatively, every 3 months for the first year, every 4 months during the second and third years, and every 6 months thereafter.

Senior authors (LC, DD, BS) clinically evaluated all the cases, considering pain, functional ability, walking ability, and gait. These data were collected according to the modified 30‐point scoring system of the Musculo Skeletal Tumor Society [22] at 3, 6, and 12 months than at last alive follow‐up. The evaluations were expressed as a percentage of the optimal score of 30, using the revised 30‐point functional evaluation system. Complications were categorized according to the Henderson et al. [23] classification as follows: type I (soft tissue failure), type II (aseptic loosening), type III (breakage), type IV (infection), and type V (local recurrence).

### 2.7 Primary and Secondary Study Outcomes

Our primary study goal was to evaluate the cumulative incidence of reoperation for any reason after pelvic resection and reconstruction with a 3D‐printed custom‐made prosthesis in patients with a primary bone tumor. We documented the reasons for reoperation, including complications such as instability, aseptic loosening, implant breakage, local recurrence, or infection, and recorded the time until the first reoperation, whether it involved removal of the reconstruction or not. Reoperations were categorized as any additional surgeries required after the initial procedure due to complications, regardless of whether the original construct was retained. We also detailed the timing of these reoperations.

Our secondary study objectives include evaluating the functional outcomes of the reconstruction, assessing the oncological adequacy of resections performed with cutting guides for local disease control, and examining the fit between the bone and prosthetic interface. We used the revised 30‐point functional evaluation system of the MSTS scores [24] as a percentage of the best possible score of 30. We characterized the status of the hip by considering pain, function, walking ability, and gait. Two authors (DD, BS) visited all patients and collected data on their functional abilities. MSTS scores were collected at 12 and 24 months, and then at the last outpatient check. In the event of implant removal or hindquarter amputation, the MSTS scores were collected at the last check before surgery.

Axial CT scans were analyzed to assess prosthesis–host integration, using criteria such as the absence of a radiolucent line at the bone–prosthesis interface, the presence of a bridging callus across three of the four cortices, and the continuity of the bone–prosthesis margin within 9 months post‐surgery. The senior authors (DD, BS, LC) reviewed and confirmed the results of all CT images.

### 2.8 Ethical Approval

The ethical committee of the institution approved the prospective data collection (General protocol no. EM468/2021_47/2014/Oss/IOR_EM1), and informed consent was obtained from each patient. This study was conducted in accordance with the Declaration of Helsinki.

### 2.9 Statistical Analysis

In our study, we presented continuous variables using the median and range, while categorical variables were expressed as percentages and frequencies. We calculated cumulative incidence curves to estimate the likelihood of discontinuation due to competing events, such as patient death. All statistical analyses were performed with IBM SPSS Statistic 21.0 (IBM Corp, Armonk, NY). The primary outcome was how long the reconstruction lasted before any further surgery, and the secondary outcome concerned the duration until a reoperation was necessary. We tracked each participant until their last follow‐up or until their death, with a significance level set at *p* = 0.05.

## 3 Results

### 3.1 Reoperations, Complications, and Reconstruction Removal

The cumulative incidence of reoperation after the first surgery was 46% (95% confidence interval: 26%–66%). The leading reason for additional surgeries was delayed wound healing (21%; *n* = 5), followed by local disease recurrence (16%; *n* = 4), deep infection (8%; *n* = 2), and dislocation (4%; *n* = 1). The mean time to reoperation was 14 ± 16 months, with 55% occurring within 3 months post‐surgery. Although the reoperation rate was higher among patients who underwent total hemipelvis reconstruction (P1–P2–P3 areas) compared to those with partial hemipelvis reconstruction (P1–P2 or P2–P3), this difference was not statistically significant.

The cumulative incidence of implant removal was 21% (95% confidence interval: 5%–37%; *n* = 5). The median time to implant removal was 17 months. Deep infection was the most common cause for implant removal (three patients), followed by local recurrence (two patients). Patients with recurrence and one with deep infection underwent hindquarter amputation, while in the remaining two patients, the implant was replaced with an antibiotic‐loaded cement spacer. Once the infection was controlled, a flail hip procedure was performed.

The cumulative incidence of postoperative complications not requiring further surgery was 33% (95% confidence interval: 14%–52%; *n* = 8). Aseptic loosening was observed in two patients (8%). It occurred at the interface between the pubic symphysis and the prosthesis, resulting in the breakage of the fixation screw. No loosening was observed at the sacral or iliac area and the prosthesis. Surgical intervention was not carried out due to the absence of symptoms and because the stability of the implant was not compromised. No hardware breakage was observed during the observation time. Five out of eight experienced common peroneal nerve palsy, and one had femoral nerve palsy, affecting both sensory and motor branches post‐surgery. The patient with femoral nerve palsy fully recovered, whereas the patients with common peroneal nerve palsy suffered permanent effects.

### 3.2 Disease Control and Implant Evaluation

Histologically negative margins (R0) were achieved at both bone resection sites in all patients. Soft tissue margins were classified according to the Enneking surgical system [25], with 96% considered wide and 4% marginal. All patients underwent surgery using a custom‐made iliac cutting guide. A pubic branch cutting guide was designed in 20 out of 24 cases (83%), as the remaining 4 cases (17%) required resection at the level of the pubic symphysis. Among the 20 patients who received a pubic branch guide, disease progression relative to the preoperative plan was noted intraoperatively in two cases. In both instances, the osteotomy line was manually extended beyond the guide to achieve appropriate oncologic margins. Postoperative CT scans demonstrated an adequate fit between the implant and the host bone at the level of the iliac osteotomy in all cases. At the sacroiliac interface, a gap greater than 1 mm between the prosthesis and the bone was observed in seven cases (29.1%). In two cases (8.3%), the osteotomy was performed beyond the preoperatively planned line.

Local recurrence was observed in five patients (21%) at a median time of 5 months post‐surgery; four of these patients had wide surgical margins at histology, while one had wide bone margins but marginal soft tissue margins. Three patients underwent hindquarter amputations, one surgical excision of the recurrence (hindquarter amputation was carried out for subsequent deep infection), and one underwent palliative radiation therapy. Three out of five patients succumbed to disease progression following recurrence: one with osteosarcoma and another with dedifferentiated chondrosarcoma died 41 and 67 months after surgery, respectively, due to metastatic disease. Another patient, initially presented with lung metastasis, experienced a recurrence 2 months post‐surgery and died 1 month later.

### 3.3 Functional Outcome

The mean MSTS scores at 12 months were available for 21 out of 24 patients (87.5%), at 24 months for 16 patients (66.6%), and at the final follow‐up for 17 patients (70%). Mean follow‐up duration was 66.4 months (range 13–137 months). The mean scores were 25.1 out of 30 points (83.7%; range 53%–100%) at 12 months, 25.2 out of 30 points (84%; range 37%–100%) at 24 months, and 25.1 out of 30 points (83.7%; range 33%–100%) at the final follow‐up.

## 4 Discussion

This study was not designed to quantify surgical accuracy or to compare techniques, but rather to document the feasibility and oncological/functional outcomes of 3D‐assisted pelvic reconstructions in a consecutive clinical series. The treatment of pelvic bone tumors, necessitating complete resection of the acetabulum and surrounding areas, poses significant challenges in maintaining pelvic continuity and long‐term hip functionality. Utilizing custom 3D‐printed prostheses and surgical guides offers a flexible approach to reconstruction, effectively addressing various defects resulting from tumor removal [26, 27, 28, 29, 30]. Surgery involving these methods carries a significant risk of complications, including infection, hardware failure, and aseptic loosening, occurring in 30%–65% of cases [31].

Our study aims to explore: (1) What is the cumulative incidence of reoperation for any reason following pelvic resection and reconstruction with a custom‐made 3D‐printed prosthesis involving the acetabulum in patients with primary bone sarcoma, and what factors contribute to an increased risk of reconstruction failure? (2) Does the use of 3D custom‐made cutting guides, combined with a 3D custom‐made hemipelvis prosthesis, ensure the attainment of safe resection margins and allow for anatomical reconstruction with optimal fit at the bone–prosthesis interface? (3) What were the observed outcome scores as measured by the MSTS score? Additionally, how do the type of resection and the volume of the primary bone sarcoma affect the outcomes in relation to the type of reconstruction?

### 4.1 Limitations

We recognize several limitations in our study. One drawback of this study is the limited number of participants. Also, we included heterogeneous diagnoses in this study. Despite this, we consider the sample size adequate to support our conclusion that discontinuing this procedure is warranted. Additionally, the retrospective nature of the study could have heightened the possibility of selection bias. Typically, selection bias might exaggerate the perceived benefits of a new method. The small scale of the study precluded a gender‐based analysis (boys vs. girls), and it is imprudent to assume that results from a mixed‐gender group are applicable to each gender individually. While we do not anticipate that gender significantly influenced the results, its exact impact remains unclear. It should also be noted that this procedure was performed over the years by the same team of surgeons, which is a valuable aspect as it minimizes potential variations in surgical technique that could occur if multiple surgeons were involved. This study lacks a control group treated with traditional reconstruction techniques, which limits our ability to directly compare outcomes. However, given the rarity of pelvic sarcomas and the heterogeneity of resection types, prospective multicenter registries may represent a more feasible and ethically appropriate path for future comparative analyses.

### 4.2 Reoperations, Complications, and Reconstruction Removal

Pelvic surgery carries a substantial risk of complications that may require additional interventions. In our cohort, we observed a complication rate of 46%, which falls within the range reported in the specialized literature for this type of surgery. However, it is crucial to acknowledge the difficulty of comparing our results with those of other studies, as the existing literature on surgeries involving custom 3D‐printed pelvic prostheses remains limited, and patient populations are highly heterogeneous (Table 2).

**TABLE 2:** Summary of major studies on pelvic reconstruction using 3D‐printed custom‐made prostheses following tumor resection of the pelvis.

Study	Number of patients	Cutting guide and implant	Follow‐up months (range)	Reoperation rate patients (%)	Complications					Mean MSTS scores XX/30 (%)
					Surgical wound difficulties (%)	Deep infection (%)	Aseptic loosening (%)	Hip dislocation (%)	Local recurrence (%)	
Dai et al. [37]	10	NoCG/CMP	34	2 (20%)	3 (30%)	0	1 (10%)	2 (20%)	3 (30%)	NR
Sun et al. [34]	16	NoCG/CMP	36 (23–62)	5 (31%)	0	1 (6%)	0	3 (19%)	3 (19%)	21/30 (70%)
Holzapfel et al. [33]	56	CG/CMP	66 (1–270)	27 (48%)	0	14 (25%)	3 (5%)	11 (20%)	10 (18%)	18/30 (60%)
Liang et al. [7]	35	NoCG/3DPP	20.5 (6–30)	9 (25.7%)	7 (20%)	0	0	2 (6%)	—	22.7 (75.6%)
Wang et al. [32]	13	3DCG/3DPCP	27 (24–31)	2 (15%)	2 (15%)	0	0	0	0	22/30 (73.3%)
Liu et al. [39]	38	3DCG/3DPCP	30 ± 2.9	—	0	0	4 (11%)	4 (11%)	9 (24%)	22/30 (73.3%)
Current study	24	3DCG/3DPCP	60 (4–135)	11 (46%)	5 (21%)	2 (8%)	2 (8%)	1 (4%)	4 (16%)	25.1/30 (83.7%)

*Note*: The MSTS score is recorded as XX of 30, where “XX” indicates the specific value on a 30‐point scale.

Abbreviations: 3DCG, 3D cutting guide; 3DPCP, 3D‐printed custom prosthesis; 3DPP, 3D‐printed prosthesis; CG, cutting guide; CMP, custom made prosthesis; NoCG, no cutting guide.

In our series, delayed wound healing occurred in 21% of patients. For comparison, Wang et al. [32] reported a 15% rate of delayed wound healing in a series of 13 patients treated with a custom‐made 3D‐printed pelvic prosthesis, without mentioning other complications. Conversely, our deep infection rate was 8%, which is notably lower than the 13%–30% range commonly reported in similar contexts [33, 34, 35, 36, 37, 38]. Despite this, the overall reoperation rate (46%) remains on the higher end of the reported spectrum. This may be partly attributed to the complexity of the procedures, particularly those involving extensive acetabular and pubic resections, which are inherently associated with higher technical risk.

Additionally, the absence of a control group treated with conventional methods limits our ability to isolate the specific contribution of 3D‐printed technology to the observed outcomes. While we believe that 3D printing enhances anatomical reconstruction and supports prosthetic alignment, it does not eliminate the multifactorial risks inherent to pelvic sarcoma surgery. Notably, deep infection—despite its relatively low incidence in our series—remained the leading cause of implant removal, accounting for three cases.

We observed a 4% dislocation rate, which is consistent with reported rates in the literature, ranging from 0% to 20% [33, 34, 35, 36, 38, 39, 40]. Dislocation typically results from compromised muscle function following extensive resection. In most instances, the gluteus medius and minimus gluteus muscles were resected, allowing the gluteus maximus origin to be sutured more anteriorly onto the abdominal muscles. This modification is an important step to increase the stability of the hip [41, 42].

**FIGURE 2:**
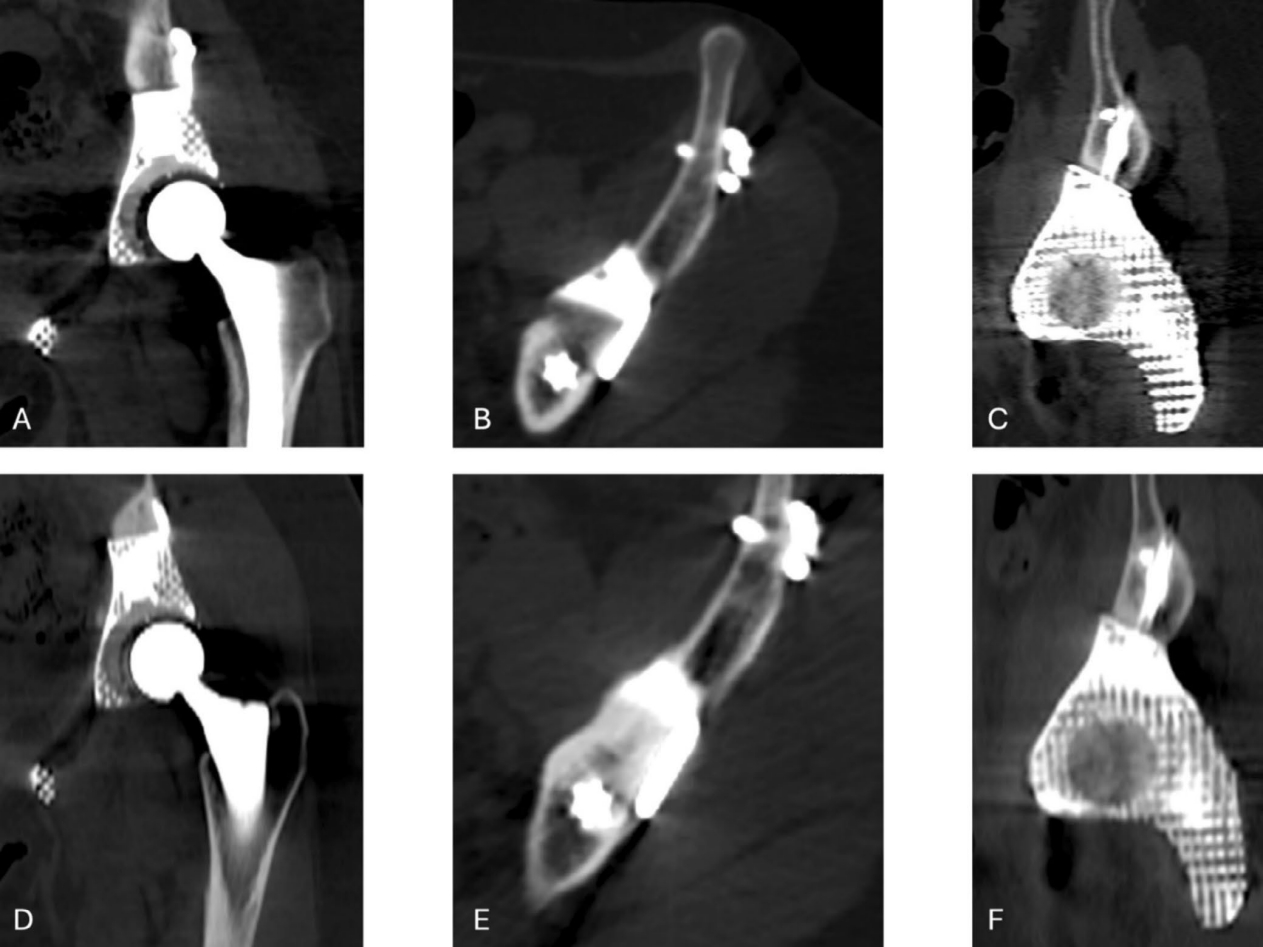
Postoperative CT images in (A) coronal, (B) axial, and (C) sagittal views show the interface between the implant and the host bone. A radiolucent line is visible along the bone–prosthesis contact surface. CT images in (D) coronal, (E) axial, and (F) sagittal projections at 5‐year follow‐up demonstrate the absence of the radiolucent line and the presence of newly formed bone, with clear evidence of bone ingrowth around the implant, indicating stable and long‐term osteointegration.

The only implant‐related complication observed in our cohort was aseptic loosening, which occurred in 8% of cases, exclusively at the site of pubic symphysis arthrodesis, and did not require revision surgery. This incidence represents one of the lowest rates reported in the literature [35, 36, 37, 39, 41]. All patients who developed aseptic loosening had received the initial implant design, in which fixation was achieved using one or two trans‐pubic screws crossing the contralateral ischiopubic ramus. This configuration resulted in screw breakage and subsequent aseptic loosening at mid‐term follow‐up. To address this complication, the prosthetic design was revised: trans‐osseous screws were replaced with a small fixation plate secured superiorly to the contralateral pubic ramus using two cortical screws. None of the patients treated with this modified design have shown signs of aseptic loosening to date. No evidence of loosening was observed at the iliac or sacral sites. Fixation to the iliac bone was achieved using custom‐designed flanges with multiple screw holes, allowing for stable and secure anchorage. In the sacral region, stability was ensured using long, obliquely oriented flanges and screws directed toward the sacral body, providing robust and reliable fixation. Additionally, the trabecular titanium structure of the prosthetic surfaces promotes bone ingrowth, further enhancing implant stability over time. This is radiologically confirmed by follow‐up CT scans showing the disappearance of the radiolucent line and the presence of newly formed bone along the bone–implant interface (Figure 2). Successful osteointegration at the iliac or sacral site is essential for ensuring optimal implant stability.

Key factors influencing osteointegration include the precise match between the osteotomy line and the prosthesis, ensured by the 3D cutting guide, the porous structure of the 3D‐printed prosthesis that promotes bone integration, and the orientation of custom ilio‐sacral screws, which enhance the mechanical stability of the acetabular region. The lack of hardware failure further supports these findings. However, we acknowledge that this study was not designed to quantitatively evaluate the surgical precision of 3D‐printed guides or implants. Although we included radiological criteria such as the absence of gaps > 1 mm and signs of bone ingrowth on CT scans, these indicators remain indirect and descriptive. Further studies using validated intraoperative navigation systems or 3D spatial deviation analysis are needed to assess resection accuracy more objectively. The incidence of other complications, including nerve palsy, falls within the expected range for this type of surgery.

### 4.3 Disease Control and Implant Evaluation

We observed a local recurrence rate of 21%, which is comparable to rates reported in the literature, ranging from 20% to 50% [26, 43]. An adequate bone margin (R0) was achieved in 100% of patients at the bone resection site, whereas an R1 margin was observed in 96% of cases at the soft tissue site.

Among patients who experienced recurrence, four had an R0 margin, while one had an R1 in the soft tissue. However, the evaluation of soft tissue margins relies primarily on preoperative MRI imaging and the surgeon's experience. Intralesional resection and positive margins identified through histological examination were associated with local recurrence and poor outcomes. These findings are consistent with the 82%–98% rate of clear margins reported in the literature [7, 26, 28, 41, 44]. The cutting guides proved consistently accurate when used for iliac resections. However, in two cases involving the ischiopubic branch, intraoperative findings revealed that the extent of the disease was greater than anticipated in the preoperative planning. As a result, the osteotomy of the ischial branch was performed manually, extending the resection beyond the area defined by the guide. However, this intraoperative adjustment did not compromise prosthesis implantation or final stability, as the iliac osteotomy was accurately performed and ensured proper matching and fixation of the implant.

This confirms that the use of patient‐specific, custom 3D‐printed cutting guides is an effective strategy that enables surgeons to achieve adequate bone resection margins during surgery. Furthermore, the time required for cutting guide planning and manufacturing (ranging from 3 to 6 weeks) does not appear to have a negative impact on surgical radicality compared to other techniques.

### 4.4 Functional Outcome

We observed a mean MSTS score of 83.7% at the end of follow‐up. These data are consistent with the literature, which reports a range of 40%–80% [28, 45, 46]. These results are superior to biological techniques such as bone allografts, which have been reported to achieve a mean MSTS score between 60% and 70% [4]. While biological reconstruction has the theoretical potential for permanent consolidation with the host bone, prosthetic reconstruction enables early mobilization, immediate and long‐term stability, and satisfactory cosmetic and functional outcomes [11, 34, 41]. Although this study did not include quantitative measurements of surgical precision—such as spatial deviation or RMS error between planned and executed resections—we applied a clinically validated workflow based on preoperative simulation, physical 3D models, and intraoperative verification. These elements enabled a high degree of control over guide positioning and implant fit, and allowed for minor adaptations during surgery to accommodate intraoperative findings. Such flexibility is inherent to the CAD‐based design process and offers a pragmatic approach in complex anatomical scenarios. This methodology may form the basis for future prospective studies incorporating 3D quantitative validation metrics, as described in recent experimental protocols [21]. The 3D printing technology integrates the advantages of both approaches [7, 34, 37]. We observed that the MSTS score reached 83.7% as early as 1 year postoperatively and remained consistently high until the end of follow‐up. The high MSTS scores observed may be attributed to the type of reconstructive approach adopted. In fact, the use of 3D‐printed custom‐made prostheses allows for accurate restoration of hip biomechanics, which likely contributes to the favorable functional outcomes. However, further studies with larger cohorts and longer follow‐up are nonetheless warranted to better investigate and confirm the functional benefits associated with this reconstructive approach.

## 5 Conclusion

The integration of 3D‐printed custom cutting guides with personalized 3D‐printed prostheses for reconstructing tumor‐induced bone defects in sarcoma patients has ushered in a new era of limb salvage surgery. The porous structure of these implants demonstrates excellent histocompatibility, promoting bone and soft tissue ingrowth. The combination of a porous bone–prosthesis interface, precise anatomical fit, and continuous force transmission contributes to improved limb function and manageable complication rates. Additionally, these implants facilitate a quicker return to function in the postoperative period. However, further research is required to objectively assess their long‐term durability and complication rates before they can be widely adopted in routine clinical practice.

## Author Contributions

Study conception and design: Dr. Benedetta Spazzoli, Dr. Luca Cevolani. Acquisition of data: Dr. Benedetta Spazzoli. Analysis and interpretation of data: Dr. Luca Cevolani, Dr. Paolo Spinnato. Drafting of manuscript: Dr. Luca Cevolani, Dr. Benedetta Spazzoli. Critical revision: Prof. Davide Maria Donati, Dr. Eric Lodewijk Staals, Dr. Laura Campanacci, Dr. Costantino Errani, Massimiliano De Paolis, Giuseppe Bianchi.

## Ethics Statement

Approval was obtained from the ethics committee of the Rizzoli Institute. The procedures used in this study adhere to the tenets of the Declaration of Helsinki.

## Consent

Informed consent was obtained from all individual participants included in the study.

## Conflicts of Interest

The authors declare no conflicts of interest.

## Data Availability

Raw data were generated at the Rizzoli Institute. Derived data supporting the findings of this study are available from the corresponding author upon request.
